# Partial silencing of fucosyltransferase 8 gene expression inhibits proliferation of Ishikawa cells, a cell line of endometrial cancer

**DOI:** 10.1016/j.bbrep.2020.100740

**Published:** 2020-02-12

**Authors:** Hana Shimoyama, Toshiaki K. Shibata, Masahiko Ito, Tomoaki Oda, Toshiya Itoh, Mari Mukai, Madoka Matsuya-Ogawa, Masashi Adachi, Hirotake Murakami, Takeshi Nakayama, Kazuhiro Sugihara, Hiroaki Itoh, Tetsuro Suzuki, Naohiro Kanayama

**Affiliations:** a Department of Obstetrics and Gynecology, Hamamatsu University School of Medicine, 1-20-1 Handayama, Higashi-ku, Hamamatsu, Shizuoka, 431-3192, Japan; b Department of Virology and Parasitology, Hamamatsu University School of Medicine, 1-20-1 Handayama, Higashi-ku, Hamamatsu, Shizuoka, 431-3192, Japan; c Department of Faculty of Medical Technology, Fujita Health University, 1-98 Dengakugakubo, Kutsukake-cho, Toyoake, Aichi, 470-1192, Japan

**Keywords:** Endometrial endometrioid carcinoma, Fucosyltransferase 8, Ishikawa cells, Cell proliferation, xCELLigence

## Abstract

Endometrial cancer is the most common gynecologic malignancy and is associated with increased morbidity each year, including young people. However, its mechanisms of proliferation and progression are not fully elucidated. It is well known that abnormal glycosylation is involved in oncogenesis, and fucosylation is one of the most important types of glycosylation. In particular, fucosyltransferase 8 (FUT8) is the only FUT responsible for α1, 6-linked fucosylation (core fucosylation), and it is involved in various physiological as well as pathophysiological processes, including cancer biology. Therefore, we aimed to identify the expression of FUT8 in endometrial endometrioid carcinoma and investigate the effect of the partial silencing of the FUT8 gene on the cell proliferation of Ishikawa cells, an epithelial-like endometrial cancer cell line. Quantitative real-time PCR analysis showed that FUT8 gene expression was significantly elevated in the endometrial endometrioid carcinoma, compared to the normal endometrium. The immunostaining of FUT8 and *Ulex europaeus* Agglutinin 1 (UEA-1), a kind of lectin family specifically binding to fucose, was detected endometrial endometrioid carcinoma. The proliferation assay showed FUT8 partial knockdown by transfection of siRNA significantly suppressed the proliferation of Ishikawa cells, concomitant with the upregulation in the gene expressions associated with the interesting pathways associated with de-ubiquitination, aspirin trigger, mesenchymal-epithelial transition (MET) et al. It was suggested that the core fucosylation brought about by FUT8 might be involved in the proliferation of endometrial endometrioid carcinoma cells.

## Highlights

• Fucosyltransferase 8 gene expression is elevated in the tissues affected by endometrial endometrioid carcinoma.
• Fucosyltransferase 8 protein is specifically detected in the glands affected by endometrial endometrioid carcinoma.
• Silencing of fucosyltransferase 8 suppressed the proliferation of Ishikawa cells, an endometrial cancer cell line.
• These results suggest that fucosyltransferase 8 might be involved in the proliferation of endometrial endometrioid carcinoma.

## 1 Introduction

Endometrial cancer is one of the most common gynecologic malignancies. In the United States, there were an estimated 54,870 newly diagnosed cases and 10,170 deaths in 2016 [1]. The associated morbidity, including among young people, increases each year. Endometrial endometrioid carcinoma is the most common histologic type of endometrial cancer and comprises 80% of all cases [2,3]. The main risk factor for endometrial endometrioid carcinoma is long-term exposure to excess endogenous or exogenous estrogen without adequate opposition by a progestin [4]. However, its mechanism of proliferation is not fully elucidated; in particular.

Carbohydrate antigens, such as CA19-9 and CA125, are established tumor markers for diagnosing and treating various kinds of cancers, including endometrial cancer because the structure of the carbohydrate chain usually changes in cancer cells [5]. Indeed, abnormalities of glycosylation, the process by which carbohydrate chains are synthesized, are frequently observed in cancer cells and cancer tissues [[6], [7], [8], [9]]. Furthermore, glycosylation and the resultant changes of the carbohydrate chain occur as a result of cellular oncogenesis and are involved in cancer cell progression, metastasis, intercellular contact, epithelial-mesenchymal transition, and more [[10], [11], [12]].

Among the numerous kinds of glycosylation, fucosylation is one of the most physiologically pivotal types and is controlled by fucosyltransferase (FUT). FUT transfers fucose from GDP-fucose to glycoconjugates, such as glycoproteins and glycolipids. Among the FUT families, FUT8 is the only enzyme responsible for α1, 6-linked fucosylation (core fucosylation) by adding fucose to the innermost GlcNAc residue of an N-linked glycan. Recently, increasing evidence has supported the crucial involvement of core fucosylation by FUT8 in carcinogenesis and cancer progression [[13], [14], [15]].

Human protein atlas described that gene and protein expression of FUT8 was elevated in endometrial endometrioid carcinoma and its high expression was associated with increased mortality [16] Wang et al. also reported that fucosyltransferase activity was significantly increased in endometrial endometrioid carcinoma compared to normal endometrium [17].

In the present study, we hypothesized that FUT8 was upregulated in endometrial endometrioid carcinoma and regulated its proliferation. The specific objectives of the present study were to investigate 1) gene expression of FUT families in the tissues of a normal endometrium and endometrial endometrioid carcinoma, 2) tissue localization of FUT8 and *Ulex europaeus* Agglutinin 1 (UEA-1), a kind of lectin family specifically binding to fucose, in a normal endometrium and endometrial endometrioid carcinoma, 3) expression of FUT8 in Ishikawa cells, an endometrial cancer cell line, 4) the effects of partial silencing of the FUT8 gene on the proliferation of Ishikawa cells, and 5) the effects of partial silencing of the FUT8 gene on gene expression patterns by microarray analysis.

## 2 Materials and methods

### 2.1 Patients and resources

Normal endometrial tissues and endometrial endometrioid carcinoma were obtained from patients who underwent hysterectomy at the Department of Obstetrics and Gynecology, Hamamatsu University Hospital between 2016 and 2017 due to gynecological diseases or endometrial endometrioid carcinoma. Written informed consent was obtained from each patient after a full explanation of the study. Patients’ backgrounds are summarized in Table 1. We excluded patients who received radiation therapy or neoadjuvant chemotherapy before surgery.

**Table 1:** Patients’ backgrounds.

Characteristic	All patients with endometrioid carcinoma (EC) (n = 13)	
	n	%
Age at diagnosis (years)		
<65	6	46.2
≧65	7	53.8
Para (times)		
0	3	23.1
1	3	23.1
2	7	53.8
FIGO stage		
Stage I + II	9	69.2
Stage III + IV	4	30.8
Grade		
G1	6	46.1
G2	5	38.5
G3	2	15.4
Myometrial invasion		
≤1/2	7	53.8
>1/2	6	46.2
Lymph node metastasis		
N0	10	76.9
N1-3	3	23.1
ER expression		
Positive	13	100
Negative	0	0
PgR expression		
Positive	12	92.3
Negative	1	7.7

G1–Less than 5% solid growth patterns; G2–6 to 50% solid growth patterns.

G3–Greater than 50% solid growth patterns.

### 2.2 Quantitative real-time PCR

The total RNA of tissues and cells were extracted using RNeasy Lipid Tissue Mini Kit (QIAGEN, Hilden, Germany) Then, cDNA was synthesized using ReverTra Ace® qPCR RT Master Mix (TOYOBO CO., LTD, Japan). The resultant cDNA was amplified using THUNDERBIRD®SYBR® qPCR Mix (TOYOBO CO., LTD, Japan). Quantitative real-time PCR reactions were performed using the StepOnePlus ™ real-time PCR system (Thermo Fisher Scientific, MA, USA) and SYBR Green Method. ACTB acted as the endogenous control. The primer sequences (Fasmac Co., Ltd, Japan) for quantitative real-time PCR are listed in Table 2. The sizes of PCR products were confirmed by electrophoresis using 2% agarose gels.

**Table 2:** List of primers for quantitative real-time PCR.

Primer	Sequence
FUT1-F	5′-ACGAAAAGCGGACTGTGG-3′
FUT1-R	5′-AGGCAGAGCTGACGATGG-3′
FUT2-F	5′-AGACCTTTTCTCCTTCTCTGCC-3′
FUT2-R	5′-CTGTTACTTGCAGCCCAACG-3′
FUT3-F	5′-ATCACCGAGAAGCTGTGGAG-3′
FUR3-R	5′-TGGCAGGAACCTCTCGTAGT-3′
FUR4-F	5′-AAGGCTAAATCTGCGCTTCTC-3′
FUT5-F	5′-GTGGAACCTGTCACCGGG-3′
FUT5-R	5′-GGGTGTGTTAAAAGGCCACG-3′
FUT6-F	5′-CCCTCTAGCATCTCCCAGAA-3′
FUT6-R	5′-GGGATCCATGGGTCAGAGT-3′
FUT7-F	5′-CCTATGAGGCCTTCGTGCCG-3′
FUT7-R	5′-CCTGTCACGCCAGGCAAAGA-3′
FUT8-F	5′-GCTTGGCTTCAAACATCCAG-3′
FUT8-R	5′-AATGTTCTTCAACATGCACCA-3′
FUT9-F	5′-CCATGTGGCCATGTCACTAC-3′
FUT9-R	5′-GAGCTCCTGAAGCAATTACACA-3′
ACTB-F	5′-AGTACTCCGTGTGGATCGGC-3′
ACTB-R	5′-GCTGATCCACATCTGCTGGA -3′

F, Forward primer; R, Reverse primer.

### 2.3 Immunohistochemistry of FUT8 and UEA-1

Tissue sections were deparaffinized and rehydrated using a graded series of xylene and alcohol solutions. After incubation in 3% hydrogen peroxide for 5 min to block endogenous peroxidase, they were incubated with the anti-FUT8 rabbit polyclonal antibody (Proteintech Group, Inc, IL, USA) or biotinylated UEA-1 (J-chemical, INC, Japan). After washing, they were incubated with the secondary antibody (Nichirei Histofine® Simple Stain MAX PO MULTI; NICHIREI CO., Japan; for anti-FUT8 rabbit polyclonal antibody) or Peroxidase-labeled streptavidin (NICHIREI CO., Japan; for biotinylated UEA-1) and DAB was used to acquire color development. Poorly differentiated lung squamous cell carcinoma and human branchial epithelium were used as a positive control for FUT8 and UEA-1, respectively.

The sections were imaged using a NanoZoomer 2.0 HT slide scanner and NDP.Viewer (Hamamatsu Photonics K. K., Japan). The images were presented at 25 × and 400 × magnification. The expression of UEA-1 in normal endometrium and endometrial endometrioid carcinoma was quantified by the image analysis software WinROOF ver 7.4 (MITANI CORPORATION, JAPAN) in the 4 area (each 3.88 mm^2^) of interest per slide.

### 2.4 Western blotting

Samples were collected and lysed in RIPA buffer, with the addition of 0.1% protease inhibitors. Protein concentration was quantitated using Pierce™ BCA Protein Assay Kit (Thermo Fisher Scientific, MA, USA). These proteins were separated by SDS-PAGE and electrotransferred onto polyvinylidene difluoride membranes (Immobilon-P, Millipore Merck Corporation, MA, USA). After blocking with 4% Block Ace (KAC Co., Ltd, Japan), membranes were incubated overnight at 4 °C with anti-FUT8 rabbit polyclonal antibody (Proteintech Group, Inc, IL, USA). Then, the membranes were incubated with secondary antibody (Anti-rabbit IgG HRP-linked antibody; Cell Signaling Technology, Danvers, MA). After adding ECL™ Select Western Blotting Detection Reagent (GE Healthcare UK Ltd, England), the signals were detected using ChemiDoc™ Touch Imaging System (BIO-RAD, CA, USA). GAPDH (GAPDH Rabbit Polyclonal antibody) (Proteintech Group, Inc, IL, USA) was used as an endogenous control.

### 2.5 Immunocytochemistry

First, we placed autoclaved coverslips on the 24-well plate and the Ishikawa cells were seeded into each well at a density of 1.0 × 10^5^ cells/well. After 24 h, we removed the medium and washed the coverslips with PBS. The slides were fixed with 4% formaldehyde phosphate on ice for 5 min. Then, they were blocked with 4% normal goat serum and 0.2% triton-X at room temperature for 20 min. They were incubated with the anti-FUT8 rabbit polyclonal antibody (Proteintech Group, Inc, IL, USA) at 4 °C overnight. After washing with PBS, they were incubated with the secondary antibody (Goat Anti-Rabbit IgG H&L Alexa Fluor® 594) (Abcam, UK) at room temperature for 1 h. The samples were mounted using VECTASHIELD Antifade Mounting Medium with DAPI (Vector Laboratories, INC., CA, USA) and imaged using an OLYMPUS IX83 inverted microscope (OLYMPUS CO., Japan). The images were taken at 400 × and 1000 × magnification. FUT8 was dyed fluorescent bright red and the nucleus was dyed fluorescent blue.

### 2.6 FUT8-expression plasmid transfection into Ishikawa cells

The human FUT8 cDNA was obtained from Riken (Japan) and subcloned into the pCAGneo (Wako, Japan), using the *Kpn*I and *Spe*I sites. Ishikawa cells were seeded into a 12-well plate at a density of 1.5 × 10^5^ cells/well. After 24 h, 188 ng/well of FUT8-expression plasmids were transfected into Ishikawa cells using Lipofectamine® LTX (Thermo Fisher Scientific, MA, USA). After 48 h, the expression of FUT8 was determined by Western blotting.

### 2.7 siRNA transfection into Ishikawa cells

FUT8 siRNAs (Silencer® Select Pre-designed siRNA) (siFUT8#1 and siFUT8#2) and negative control siRNA (Silencer® Select Negative Control No.1 siRNA) (siCont) were synthesized by applied Biosystems (Thermo Fisher Scientific, MA, USA). The sequences of FUT8 siRNA are as follows: SiFUT8#1 sense (5′-GAGUGAUCCUGGAUAUACAtt-3′), antisense (5′-UGUAUAUCCAGGAUCACUCca-3′), SiFUT8#2 sense (5′-CGAGUUGCUUAUGAAAUUAtt-3′), antisense (5′-UAAUUUCAUAAGCAACUCGac-3′).

Ishikawa cells were seeded into a 12-well plate at a density of 1.5 × 10^5^ cells/well. After 24 h, the siFUT8#1, siFUT8#2 or siCont (50 pmol/well) was transfected using Lipofectamine™ RNAiMAX Transfection Reagent (Thermo Fisher Scientific, MA, USA). The resultant gene and protein expression of FUT8 was determined by quantitative real-time PCR and Western blotting, respectively.

### 2.8 Cell proliferation assay using the xCELLigence Real-Time Cell Analyzer system

Cell proliferation was assessed by using the xCELLigence Real-Time Cell Analyzer (SCRUM Inc, Japan). The xCELLigence instrument uses noninvasive electrical impedance monitoring to quantify cell proliferation, morphological changes, and attachment quality in a label-free, real-time manner. In this system, impedance is converted to a parameter called the cell index (CI) and used as a monitoring index. 24 h after transfection of siFUT8#1, siFUT8#2, and siCont into Ishikawa cells, they were seeded in the chamber of E-Plate16 (SCRUM Inc, Japan) at a count of 5 × 10^3^ cells/well in triplicate. Similarly, 24 h after transfection of FUT8-expression plasmids into Ishikawa cells, they were seeded in the chamber of E-Plate16 (SCRUM Inc, Japan) at a count of 5 × 10^3^ cells/well in duplicate. After that, cell proliferation was monitored every 15 min for 160 h. Doubling time was analyzed by Real-Time Cell Analyzer software.

### 2.9 Cell invasion assay

Cell invasion assay was performed using CytoSelectTM 24-well cell invasion assay system (CELL BIOLABS, INC., CA, USA). 48 h after transfection of siFUT8#1, siFUT8#2 and siCont into Ishikawa cells, siCont, siFUT8#1 and siFUT8#2 cells suspending (2.5 × 10^5^ cells/ml) in the serum-free medium were placed in the Insert with Matrigel-coated membrane. And the lower plate was loaded with 500 μL medium containing 10% fetal bovine serum. After incubation for 24 h in a cell culture incubator, the medium was aspirated from the inside of the Insert. Cells that had not invaded the membrane were wiped out with wet swabs. On the other hand, cells that had invaded to the lower membrane were stained with Cell Stain Solution (CELL BIOLABS, INC., CA, USA), counted with a light microscope under high magnification objective, with at least three individual fields per insert.

### 2.10 Microarray analysis

After checking RNA quality, total cDNA was synthesized and purified using GeneChipTM WT PLUS Reagent Kit (Thermo Fisher Scientific, MA, USA). Then in vitro transcription and T7 RNA amplification were performed. The fragmentation and labeling of cDNA were performed using GeneChipTM Hybridization, Wash, and Stain Kit (Thermo Fisher Scientific, MA, USA). The prepared sample was hybridized, washed and stained using an automated system (GeneChipTM Scanner 3000 7G System; Thermo Fisher Scientific, MA, USA). DNA microarray experiments were performed using GeneChip Human Gene 2.0 ST Array (Thermo Fisher Scientific, MA, USA). The hybridization signal on the chip was scanned using a GeneChip 30007G scanner (Thermo Fisher Scientific, MA, USA) and processed by microarray data analysis tool in consideration of NCBI data base, which was analyzed by a software from Filgen Inc., Nagoya, Japan. The DNA microarray expression profiles were compared between siCont (n = 3) and siFUT8#2 (n = 3).

### 2.11 Statistical analysis

Data are expressed as means ± standard deviations. Significant differences between two mean values were assessed using Student's t-test or the Mann–Whitney *U* test, as appropriate. Significant differences among three mean values were assessed with Turkey–Kramer test. A *P* value less than 0.05 was regarded as significant.

### 2.12 Approval

The Ethics Committee of Hamamatsu University School of Medicine approved all procedures (approval number RI 15–309). Written informed consent was obtained from each patient after a full explanation of the study.

## 3 Results

### 3.1 FUT8 gene expression was elevated in the tissues of endometrial endometrioid carcinoma

The gene expression of FUT7 and FUT8 was significantly increased in endometrial endometrioid carcinoma tissues, compared to those of the normal endometrium (*P* < 0.05, Fig. 1F and Fig. 1G). The FUT5 gene expression was below detection sensitivity in both normal endometrial and endometrial endometrioid carcinoma tissues (data not shown). The other 6 FUT genes were expressed in both normal endometrial and endometrial endometrioid carcinoma, but there was no statistically significant difference between them (Fig. 1A, B, C, D, E, and H). The size of each PCR product was confirmed by electrophoresis with 2% agarose gel (data not shown).

**Fig. 1:**
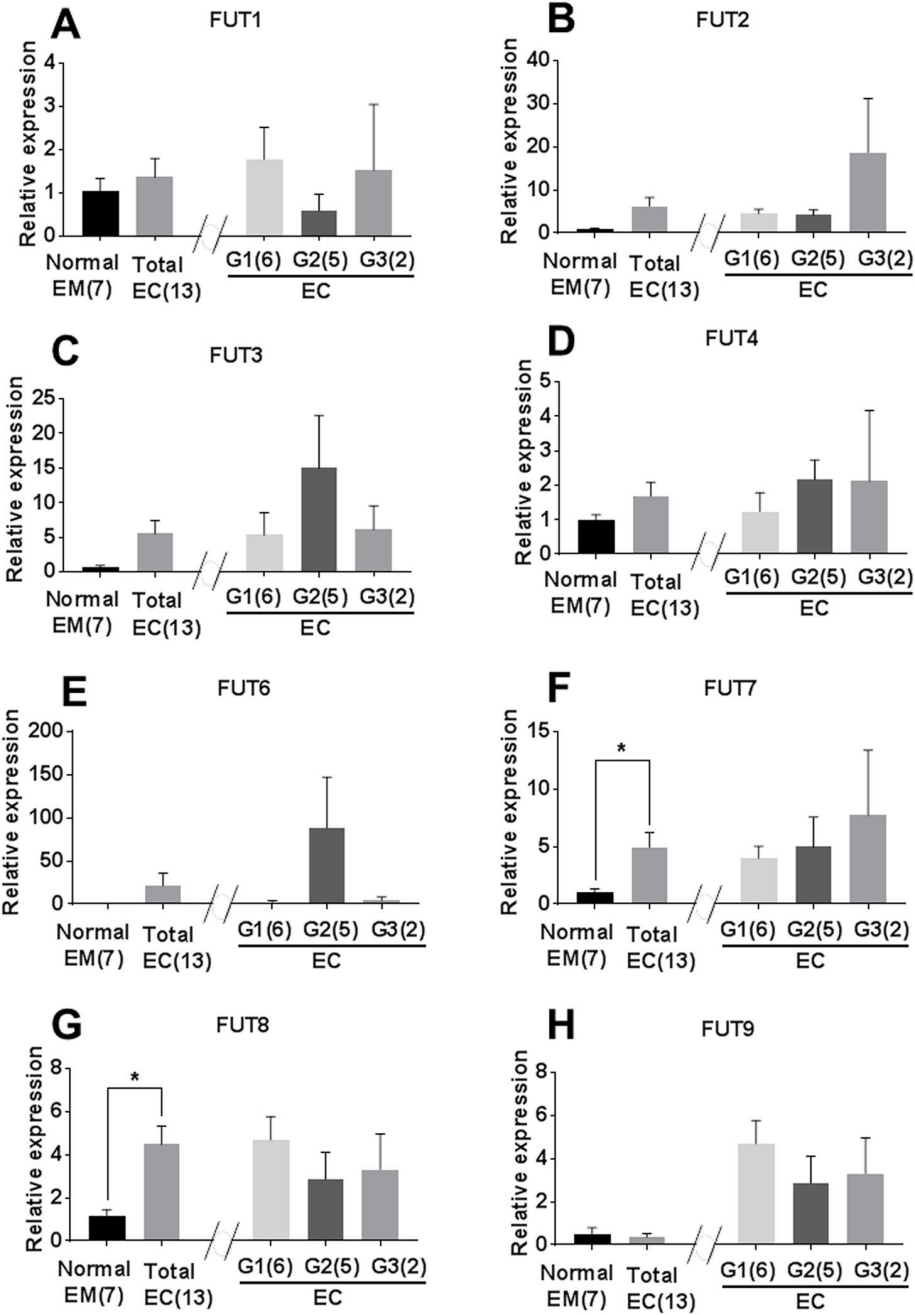
The gene expression of FUT families in normal endometrial (EM) and endometrial endometrioid carcinoma (EC) tissues. G1, G2, and G3 indicate histological classifications of EC. *; *P* < 0.05.

### 3.2 FUT8 protein was specifically detected in the gland of endometrial endometrioid carcinoma

In the normal endometrium, FUT8 protein was slightly stained in the gland of the proliferation (Fig. 2A), but not secretory (Fig. 2B) phase. In contrast, FUT8 protein was strongly and extensively stained in the gland of the endometrial endometrioid carcinoma (Fig. 2C). FUT8 immunostaining was localized in the cytoplasm of glandular epithelial cells, especially in the luminal side, and the staining patterns were dot-like, as well as diffused (Fig. 2C). FUT8 was not stained in the stromal part in either normal endometrial or endometrial endometrioid carcinoma tissues. Poorly differentiated lung squamous cell carcinoma tissue was used as a positive control for FUT8 immunostaining (Fig. 2D).

**Fig. 2:**
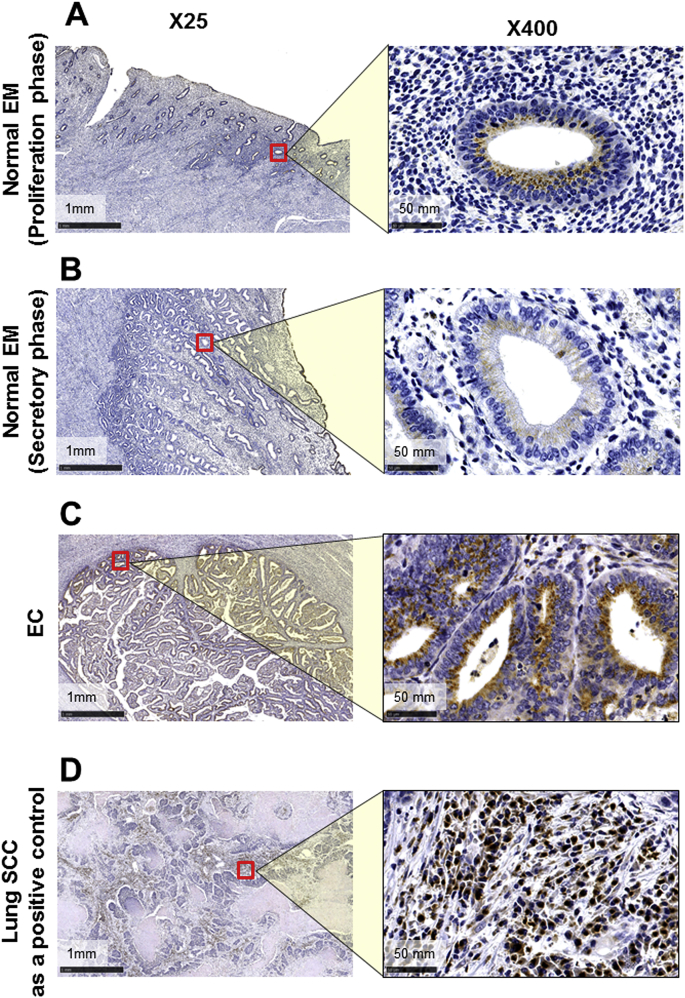
The immunohistochemical localization of FUT8 in normal endometrial tissue (EM; A, B), endometrial endometrioid carcinoma tissue (EC; C), and poorly differentiated squamous cell carcinoma lung tissue as a positive control (D). Original magnification were × 25 (left panels) and × 400 (right panels). Red squares indicate magnified areas ( × 400).

### 3.3 UEA-1 lectin expression was elevated in the tissues of endometrial endometrioid carcinoma

UEA-1 was slightly stained in the gland of the normal endometrium (Fig. 3A and Fig. 3B). In contrast, UEA-1 was strongly and extensively stained in the glandular part (Fig. 3C) as well as solid part (data not shown) of the endometrial endometrioid carcinoma. Bronchial epithelial cells were used as a positive control for UEA-1 immunostaining (Fig. 2D). The relative expression of UEA-1 was significantly increased in endometrial endometrioid carcinoma (*P* < 0.01; Fig. 3E).

**Fig. 3:**
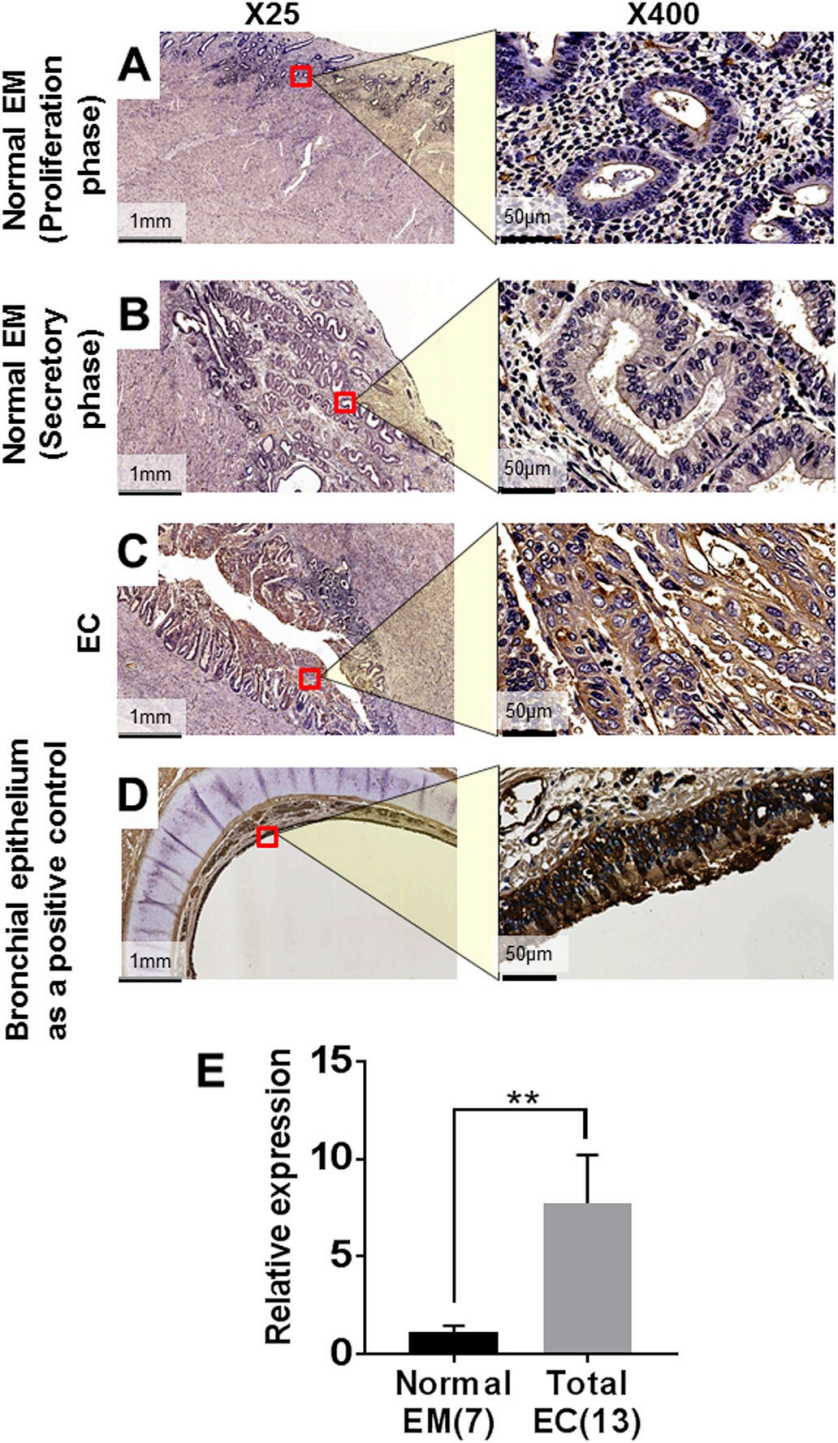
The immunohistochemical localization of UEA-1 in normal endometrial tissue (EM; A, B), endometrial endometrioid carcinoma tissue (EC; C) and bronchial epithelium as a positive control (D). Original magnification were × 25 (left panels) and × 400 (right panels). Red squares indicate magnified areas ( × 400). **; *P* < 0.01.

### 3.4 The gene and protein expressions of FUT8 in Ishikawa cells

**Fig. 4:**
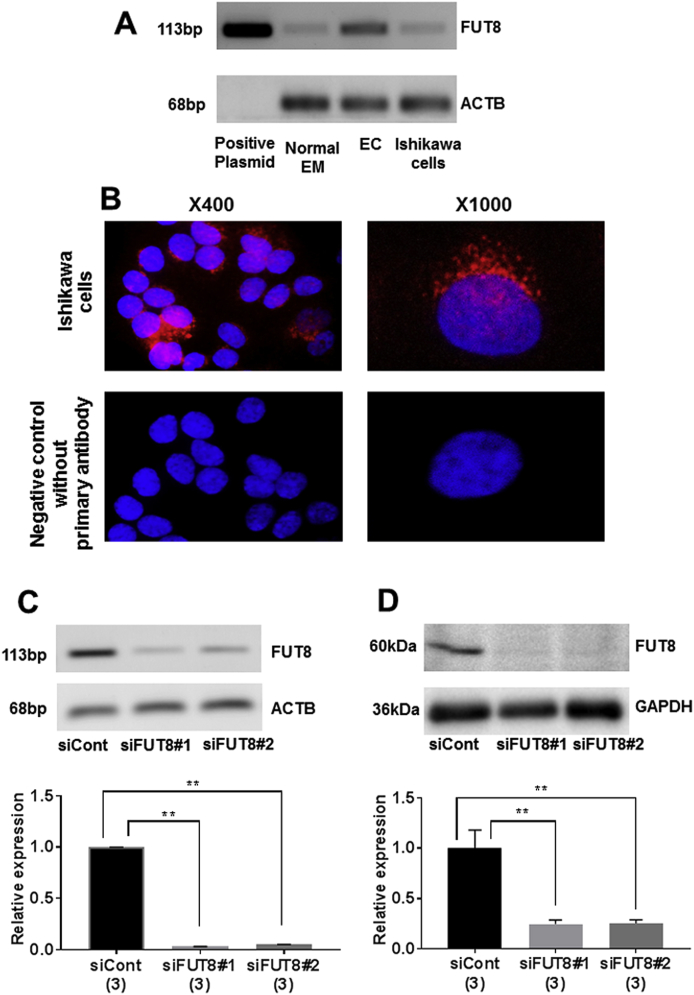
The gene and protein expression (A, B) and knockdown efficiency (C, D) of FUT8 in Ishikawa cells. Original magnification were×400 and × 1000 (B). **; *P* < 0.01. PCR products of upper panel of A and C were obtained after 28 cycles of Thermal Cycler PCR.

Quantitative real-time PCR and Western blot analysis detected gene and protein expression of FUT8 in Ishikawa cells, respectively (Fig. 4A). Immunocytochemistry also confirmed the protein expression of FUT8 in Ishikawa cells (Fig. 4B).

### 3.5 FUT8 partial knock down suppressed the proliferation of Ishikawa cells

Quantitative real-time PCR showed that transfection of siFUT8#1 or siFUT8#2 into Ishikawa cells significantly downregulated the gene expression of FUT8, compared with siCont (Fig. 4C). Knockdown efficiency of siFUT8#1 and siFUT8#2 was further confirmed by Western blot analysis (*P* < 0.01, Fig. 4D).

**Fig. 5:**
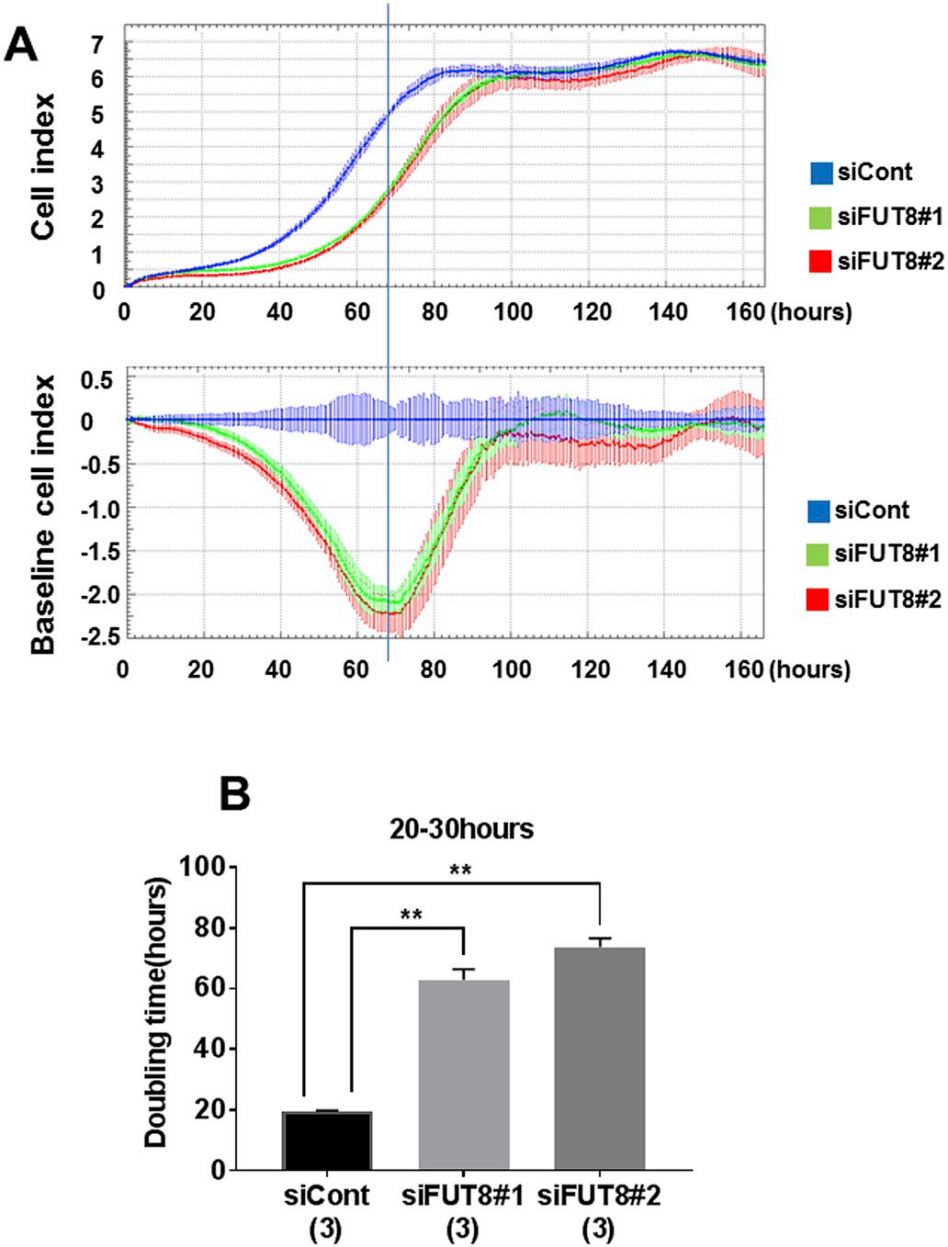
Partial knock down of FUT8 suppressed the proliferation cell index (A) and doubling time (B) of Ishikawa cells. **; *P* < 0.01.

FUT8 partial knockdown by the transfection of either siFUT8#1 or siFUT8#2 suppressed proliferation of Ishikawa cells, as indicated as CI, compared with that of siCont (Fig. 5A). Transfection of either siFUT8#1 or siFUT8#2 significantly extended the doubling time of Ishikawa cells from the start of measurement to 20 h–30 h, compared with that of siCont (*P* < 0.01, Fig. 5B).

On the other hand, overexpression of FUT8 by transfection of plasmid, confirmed by Western blot analysis (Supplementary Fig. S1A) did not affect the proliferation of Ishikawa cells (Supplementary Fig. S1B).

### 3.6 The effect of FUT8 partial knock down on the invasiveness of Ishikawa cells

We confirmed that transfection of siFUT8#1 or siFUT8#2 into Ishikawa cells significantly downregulated the gene expression of FUT8, compared with siCont in quantitative real-time PCR (data not shown). Knockdown efficiency of siFUT8#1 and siFUT8#2 was further confirmed by Western blot analysis (*P* < 0.01, Supplementary Fig. S2A).

Since lower and upper sides of chambers were filled with culture media with or without serum. We confirmed the changes of cell numbers by siFUT8#1 and siFUT8#2 transfection in both conditions. The transfection of siFUT8#1 and siFUT8#2 significantly decreased cell numbers of Ishikawa cell in the media with serum (*P* < 0.01; Supplementary Fig. S2B), but not without serum (Supplementary Fig. S2C). The net numbers of invaded cells were significantly decreased by siFUT8#1 and siFUT8#2 (*P* < 0.01; Supplementary Fig. S2D). However, there were no statistically significant differences after adjustment by cell numbers of with (Supplementary Fig. S2E) or without serum (Supplementary Fig. S2F).

### 3.7 Microarray analysis of Ishikawa cells with FUT8 partial knock down

To figure out the downstream molecular events in response to the alteration of core fucosylation, microarray analysis was performed to investigate the changes in gene expression following FUT8 silencing. The genes significantly (*P* < 0.01) upregulated compared to control were shown in Table3A. On the other hand, the genes significantly (*P* < 0.05) downregulated compared to control were shown in Table3B.

**Table 3:** Upregulation (A) and downregulation (B) of genes after FUT8 knockdown.

A						
No.	Name of pathway	Numbers of Changed genes	Total numbers of genes	Z score	P-value	Gene symbols
1	Ub-specific processing proteases_1427862	10	209	14.4	< 1 × 10^−11^	USP17L11, USP17L12, USP17L1, USP17L18, USP17L19, USP17L20, USP17L21, USP17L22, USP17L25, USP17L5
2	Deubiquitination_1427860	10	282	12.2	< 2 × 10^−10^	USP17L11, USP17L12, USP17L17, USP17L18, USP17L19, USP17L20, USP17L21, USP17L22, USP17L25, USP17L5
3	Post-translational protein modification_1268701	11	999	6.1	< 2 × 10^−6^	USP17L11, USP17L12, USP17L17, USP17L18, USP17L19, USP17L20, USP17L21, USP17L22, RAB39A, USP17L25, USP17L5
4	Metabolism of proteins_1268677	12	1562	4.7	< 2 × 10^−5^	USP17L11, USP17L12, USP17L17, USP17L18, USP17L19, USP17L20, USP17L21, USP17L22, RAB39A, USP17L25, USP17L5, MBTPS1
5	Axon guidance_83065	3	173	4.3	0.006	SEMA3C, MET, SEMA6A
6	aspirin triggered resolvin D biosynthesis_782385	1	2	15.3	0.006	PTGS2
7	aspirin triggered resolvin E biosynthesis_782386	1	2	15.3	0.006	PTGS2
8	aspirin-triggered lipoxin biosynthesis_782387	1	2	15.3	0.006	PTGS2
9	MET activates STAT3_1457803	1	3	12.4	0.008	MET
10	Semaphorin interactions_1270304	2	66	4.9	0.009	MET, SEMA6A
11	MET activates PTPN11_1457798	1	5	9.6	0.013	MET
12	MET interacts with TNS proteins_1457801	1	5	9.6	0.013	MET
13	MET Receptor Activation_1457795	1	6	8.7	0.015	MET
14	MET activates PI3K/AKT signaling_1457797	1	6	8.7	0.015	MET
15	Synthesis of 15-eicosatetraenoic acid derivatives_1270091	1	6	8.7	0.015	PTGS2
16	C20 prostanoid biosynthesis_545299	1	7	8.1	0.017	PTGS2
17	Constitutive Signaling by NOTCH1 t(7; 9)(NOTCH1:M1580_K2555) Translocation Mutant_1268885	1	7	8.1	0.017	JAG1
18	Signaling by NOTCH1 t(7; 9)(NOTCH1:M1580_K2555) Translocation Mutant_1268884	1	7	8.1	0.017	JAG1
19	Sema4D mediated inhibition of cell attachment and migration_1270306	1	8	7.5	0.019	MET
20	Synthesis of PG_1270068	1	8	7.5	0.019	PTPMT1
21	MET receptor recycling_1457804	1	9	7.1	0.021	MET
22	TNF signaling pathway_812256	2	108	3.7	0.022	JAG1, PTGS2
23	MET activates RAP1 and RAC1_1457802	1	11	6.4	0.025	MET
24	MET activates RAS signaling_1457796	1	11	6.4	0.025	MET
25	Signaling by NOTCH3_1269541	1	11	6.4	0.025	JAG1
26	Signaling by NOTCH4_1269542	1	11	6.4	0.025	JAG1
27	ATF6 (ATF6-alpha) activates chaperones_1268757	1	12	6.1	0.027	MBTPS1
28	Constitutive Signaling by NOTCH1 HD Domain Mutants_1268887	1	15	5.4	0.033	JAG1
29	InlB-mediated entry of Listeria monocytogenes into host cell_1470919	1	15	5.4	0.033	MET
30	Signaling by NOTCH1 HD Domain Mutants in Cancer_1268886	1	15	5.4	0.033	JAG1
31	Synthesis of Prostaglandins (PG) and Thromboxanes (TX)_1270088	1	15	5.4	0.033	PTGS2
32	Syndecan-1-mediated signaling events_138046	1	17	5.0	0.037	MET
33	MET activates PTK2 signaling_1457800	1	18	4.9	0.039	MET
34	Nicotinamide salvaging_1270155	1	18	4.9	0.039	PTGS2
35	Other semaphorin interactions_1270311	1	18	4.9	0.039	SEMA6A
36	Listeria monocytogenes entry into host cells_1470917	1	20	4.6	0.043	MET
37	S1P1 pathway_137937	1	20	4.6	0.043	PTGS2
38	NOTCH2 Activation and Transmission of Signal to the Nucleus_1269539	1	21	4.5	0.045	JAG1
39	Negative regulation of MET activity_1457805	1	21	4.5	0.045	MET

B						
No.	Name of pathway	Numbers of Changed genes	Total numbers of genes	Z score	P-value	Gene symbols
1	Histidine catabolism_1270162	1	8	14.1	0.006	CARNMT1
2	Small interfering RNA (siRNA) biogenesis_1269721	1	8	14.1	0.006	DICER1
3	Reactions specific to the complex N-glycan synthesis pathway_1268732	1	10	12.6	0.007	FUT8
4	eNOS activation_1270117	1	11	12.0	0.007	DDAH1
5	Glycosaminoglycan biosynthesis - keratan sulfate_82983	1	14	10.6	0.009	FUT8
6	N-glycan biosynthesis, complex type_413356	1	16	9.9	0.010	FUT8
7	Metabolism of nitric oxide_1270115	1	21	8.6	0.013	DDAH1
8	eNOS activation and regulation_1270116	1	21	8.6	0.013	DDAH1
9	MicroRNA (miRNA) biogenesis_1269720	1	23	8.2	0.015	DICER1
10	Histidine metabolism_82958	1	24	8.1	0.015	CARNMT1
11	mucin core 1 and core 2 O-glycosylation_1108786	1	24	8.1	0.015	GALNT1
12	O-glycan biosynthesis, mucin type core_413362	1	25	7.9	0.016	GALNT1
13	N-glycan antennae elongation in the medial/trans-Golgi_1268730	1	26	7.7	0.017	FUT8
14	COPI-independent Golgi-to-ER retrograde traffic_1383043	1	28	7.4	0.018	GALNT1
15	Mucin type O-glycan biosynthesis_82977	1	29	7.3	0.018	GALNT1
16	Detoxification of Reactive Oxygen Species_1270420	1	37	6.4	0.023	PRDX3
17	Histidine, lysine, phenylalanine, tyrosine, proline and tryptophan catabolism_1309221	1	43	5.9	0.027	CARNMT1
18	N-Glycan biosynthesis_82975	1	49	5.5	0.030	FUT8
19	O-linked glycosylation of mucins_1268736	1	64	4.8	0.039	GALNT1
20	Validated targets of C-MYC transcriptional activation_169351	1	77	4.3	0.047	PRDX3

## 4 Discussion

The gene expression of FUT families was identified in the normal endometrium and endometrial endometrioid carcinoma tissues. FUT8 gene expression was significantly increased in the tissues of endometrial endometrioid carcinoma compared with those of the normal endometrium (Fig. 1G), which coincided with the description of Human protein atlas [16] and the previous report [17].

FUTs enable transfer of fucose from GDP-fucose to glycoconjugates, such as glycoproteins and glycolipids. So, far, 11 kinds of FUTs of FUT1 to 11 are known, and the functions of FUT1 to 9 were previously elucidated [18]. Depending on the site of fucosylation, the FUTs are classified into α1,2 (FUT1 and FUT2), α1,3/4 (FUT3, FUT4, FUT5, FUT6, FUT7, FUT9), and α1,6 (FUT8) [19]. Among the 11 kinds of FUTs, FUT8 is the only fucosyltransferase responsible for α1,6-linked fucosylation (core fucosylation) which adds fucose to the innermost GlcNAc residue of an N-glycan [20]. Core fucosylation by FUT8 is involved in various physiological, as well as pathophysiological processes, including cancer biology [[21], [22], [23]].

Slight immunostaining of FUT8 was detected in the granular portion, on the luminal side of the gland during the proliferation phase in the normal endometrial tissue (Fig. 2A). On the other hand, it was hardly detected during the secretory phase (Fig. 2B). However, quantitative real-time PCR analysis did not show statistically significant differences in FUT8 gene expression levels between the proliferation and secretory phases of the normal endometrium (data not shown), which is probably due to very low basal levels of gene expression.

Endometrial endometrioid carcinoma consists of a glandular part and a solid part. A poorer prognosis is associated with fewer glandular parts relative to the solid parts [24,25]. Interestingly, immunostaining of FUT8 was detected in the glandular part, but not the solid part (Fig. 2C). The specific expression of FUT8 in the glandular part is also reported in cases of ovarian serous carcinoma [13]. Thyroid papillary carcinoma, which maintains a relatively glandular structure, highly expresses FUT8; however, anaplastic transformation reduces its expression [26].

UEA-1 is a lection that specifically binds fucose and positive correlation between the UEA-1 staining and myometrial and vascular invasion has been reported in endometrial carcinoma [27]. The expression of UEA-1 was significantly increased in endometrial endometrioid carcinoma compared to normal endometrium, which coincided with the previous reports [28]. Since UEA-1 expression patterns did not exactly identify the core fucosilation, we carried out immunohistochemistry of *Lens culinaris* Agglutinin (LCA), *Aleuria aurantia* lectin (AAL) and *Pholiota squarrosa* lectin (PhoSL); however, we could not obtain specific staining due to our technical limitations (data not shown).

FUT8 greatly changes the carbohydrate chain structure. For example, it was reported that the bisecting GlcNAc was added to N-glycan chain by the absence of FUT8 [29]. Bisecting GlcNAc is a GlcNAc residue in the central part of N-glycan [30], which suppresses the extension of the complicated branching of N-glycan [31,32]. Therefore, significant augmentation of FUT8 gene expression in endometrial endometrioid carcinoma strongly suggests a pivotal involvement in its biology.

Indeed, a partial knockdown of FUT8 significantly suppressed the proliferation of Ishikawa cells (Fig. 5), which was an epithelial-like endometrial cancer cell line [33], indicating a crucial role of FUT8 in their proliferation.

The present findings and evidence obtained from examining other cancers lead us to speculate that FUT8 may be involved in the regulation of cancer proliferation, specifically in the rather differentiated portions characterized by an epithelial-like glandular structure.

Increasing evidence supports the theory that core fucosylation by FUT8 influences cancer biology by regulating growth factor functions [34]. In particular, there are several reports that the abnormal fucosylation increases followed by the upregulation of TGF-β signaling [15,35]. However, our pilot study showed that partial silencing of FUT8 gene expression did not cause the significant changes in the gene expressions of TGF-β (Supplementary Fig. S3) and that microarray analysis did not detect any significant changes in the gene expressions of downstream markers of TGF-β signaling pathways, such as E-cadherin, Claudin-1, N-cadherin, α-smooth muscle actin, etc. after partial silencing of FUT8 gene expression (data not shown).

Wang X et al. reported that embryonic fibroblasts derived from FUT8-null mice significantly suppressed cell proliferation by inhibiting core fucosylation of EGFR [36] and Wang Y et al. reported that a FUT8 gene deficiency resulted in the attenuation of responses to epidermal growth factor and hepatocyte growth factor in a human liver cancer-derived cell line HepG2 [37].

Pathway analysis of microarray detected 39 activation and 20 inhibition of signaling pathways by partial silencing of FUT8 based on NCBI data base (Table 3A and 3B). The most apparent activations were observed in 2 de-ubiquitination pathways by partial silencing of FUT8, both of which was highly associated with ubiquitin specific protease 17 (USP17) families (Table3A). Shin et al. reported that overexpression of USP17 led to cell apoptosis [38]. Ramakrishna et al. demonstrated an anti-tumor activity of USP17 [39]. There have been no reports the involvement of core fucosylation in the regulation of USP17-associated pathways, as far as we know. Nevertheless, the present findings suggest a possibility that partial silencing of FUT8 may suppress cell proliferation of Ishikawa cells, at least partly, via activation of USP 17-associated pathways.

Partial silencing of FUT8 significantly elevated 3 aspirin triggered pathways (Table3A). Matsuo et al. reported that low-dose aspirin use was associated with improved survival outcomes in women with endometrial cancer [40]. Similar evidences were also reported in other cancers [41,42], although the exact mechanism has not been clarified. It is interesting to speculate that decrease of core fuosylation by partial silencing FUT8 may affect aspirin triggered pathways. There were 8 activated pathways associated with mesenchymal epithelial transition (MET) (Table3A). As far as we know, there have been no reports demonstrating a direct involvement of MET in the suppression of the progression of endometrial cancer, although it was reported that MET inhibitor suppressed the progression of several other cancers [43,44].

Decrease of FUT8 and resultant addition of bisecting GlcNAc to N-glycan may influence cancer cell proliferation. Miwa et al., reported that the presence of the bisecting GlcNAc on mammary glycoproteins reduced mammary tumor cell progression [45]. GnT-III and the bisecting GlcNAc affects cell growth and proliferation in cancer cells by suppressing the extension and the complicated branching of N-glycan [21]. Future studies will investigate the possible contribution of the changes linking carbohydrate chain structure to the FUT8-associated regulation of Ishikawa cell proliferation, especially associated with the upregulation of USP17 and/or aspirin triggered pathways.

Several investigators reported the involvement of FUT8 in the invasiveness of cancer cells [14,15]. Partial silencing of FUT8 significantly, but slightly, suppressed the net numbers of invaded Ishikawa cells (*P* < 0.01; Supplementary Fig. S2D). However, there were no statistically significant differences after adjustment of the concomitant suppression of cell proliferation (Supplementary Figs. S2E and S2F). More investigation would be necessary to clarify a possible relationship between FUT8 and cell invasiveness of Ishikawa cells.

Some investigators reported possible involvement of FUT8 in cancer adhesion, migration, invasion, and metastasis [46,47]. However, the present study featured a small patient cohort, and we could not identify any association of the levels of FUT8 expression with clinical stages of endometrial endometrioid carcinoma (data not shown). Future multicenter studies should investigate the relationship of FUT8 with clinical stage, prognosis, and prognostic factors such as myometrial invasion, lymph node metastasis, and multiple metastases, in a large cohort of patients with endometrial endometrioid carcinoma. In particular, attention should be paid to the ratio of the glandular and solid parts of tumor tissues. Care should be taken when interpreting this study's findings because the results of in vitro investigations using Ishikawa cells do not always represent the *in vivo* biology of endometrial endometrioid carcinoma.

## 5 Conclusion

In conclusion, FUT8 was expressed in endometrial endometrioid carcinoma especially in the glandular part. Partial knockdown of the FUT8 gene significantly suppresses Ishikawa cell proliferation.

## Funding

This study was supported by JSPS KAKENHI, Japan [Grant Number 18K09258] and the Hamamatsu University School of Medicine Grant-in-Aid, Japan.

## Declaration of competing interest

The authors declare no competing interests.

## Figures and Tables

**figs1:**
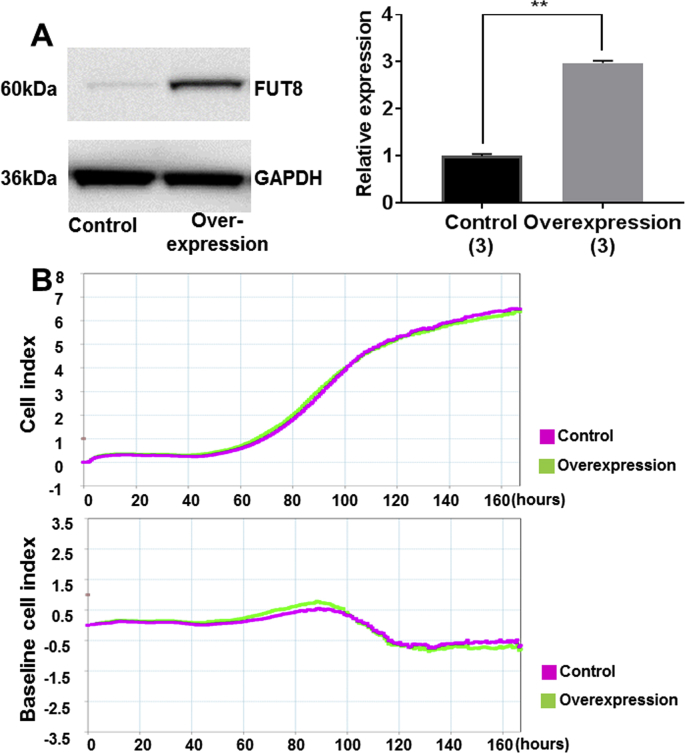


**figs2:**
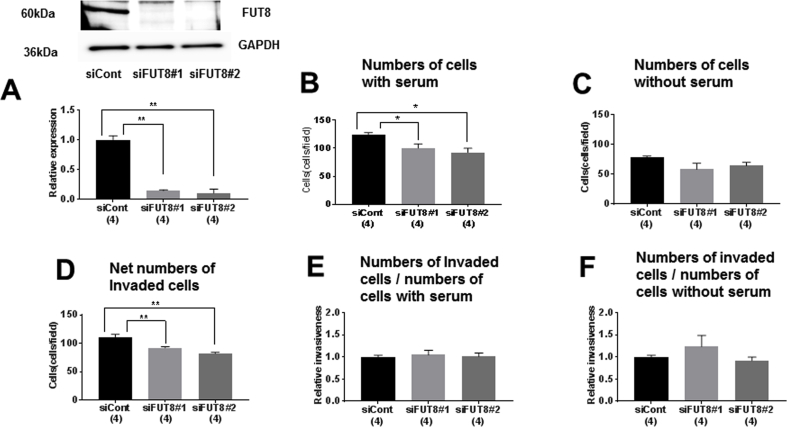


**figs3:**
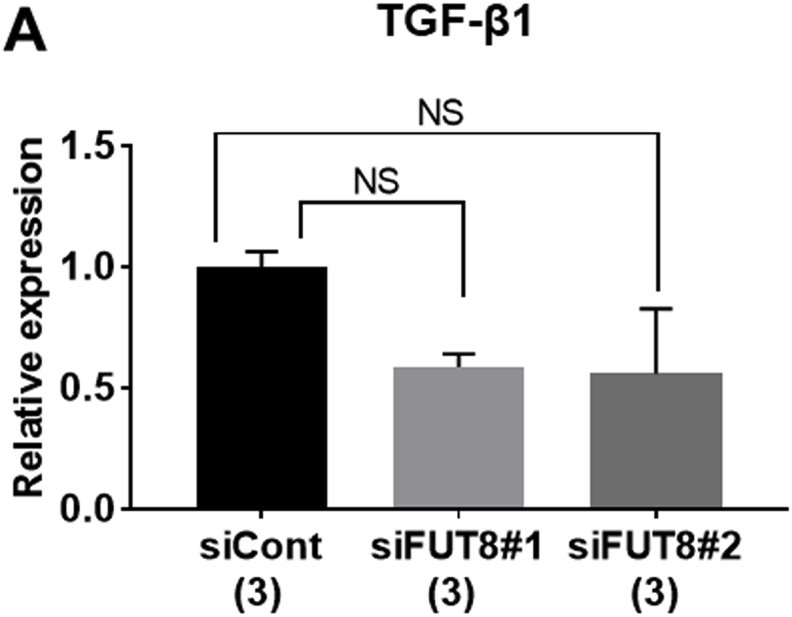


## References

[1] Siegel R.L., Miller K.D., Jemal A. (2016). Cancer statistics. CA A Cancer J. Clin..

[2] Bokhman J.V. (1983). Two pathogenetic types of endometrial carcinoma. Gynecol. Oncol..

[3] Felix A.S., Weissfeld J.L., Stone R.A., Bowser R., Chivukula M., Edwards R.P., Linkov F. (2010). Factors associated with Type I and Type II endometrial cancer. Cancer Causes Control.

[4] Brinton L.A., Berman M.L., Mortel R., Twiggs L.B., Barrett R.J., Wilbanks G.D., Lannom L., Hoover R.N. (1992). Reproductive, menstrual, and medical risk factors for endometrial cancer: results from a case-control study. Am. J. Obstet. Gynecol..

[5] Hakomori S. (1989). Aberrant glycosylation in tumors and tumor-associated carbohydrate antigens. Adv. Canc. Res..

[6] Hakomori S. (1996). Tumor malignancy defined by aberrant glycosylation and sphingo(glyco)lipid metabolism. Cancer Res..

[7] Hakomori S. (2001). Tumor-associated carbohydrate antigens defining tumor malignancy: basis for development of anti-cancer vaccines. Adv. Exp. Med. Biol..

[8] Taniguchi N., Miyoshi E., Gu J., Honke K., Matsumoto A. (2006). Decoding sugar functions by identifying target glycoproteins. Curr. Opin. Struct. Biol..

[9] Taniguchi N., Miyoshi E., Ko J.H., Ikeda Y., Ihara Y. (1999). Implication of N-acetylglucosaminyltransferases III and V in cancer: gene regulation and signaling mechanism. Biochim. Biophys. Acta.

[10] Kalluri R., Weinberg R.A. (2009). The basics of epithelial-mesenchymal transition. J. Clin. Invest..

[11] Pinho S.S., Oliveira P., Cabral J., Carvalho S., Huntsman D., Gartner F., Seruca R., Reis C.A., Oliveira C. (2012). Loss and recovery of Mgat3 and GnT-III Mediated E-cadherin N-glycosylation is a mechanism involved in epithelial-mesenchymal-epithelial transitions. PloS One.

[12] Terao M., Ishikawa A., Nakahara S., Kimura A., Kato A., Moriwaki K., Kamada Y., Murota H., Taniguchi N., Katayama I., Miyoshi E. (2011). Enhanced epithelial-mesenchymal transition-like phenotype in N-acetylglucosaminyltransferase V transgenic mouse skin promotes wound healing. J. Biol. Chem..

[13] Takahashi T., Ikeda Y., Miyoshi E., Yaginuma Y., Ishikawa M., Taniguchi N. (2000). alpha1,6fucosyltransferase is highly and specifically expressed in human ovarian serous adenocarcinomas. Int. J. Canc..

[14] Chen C.Y., Jan Y.H., Juan Y.H., Yang C.J., Huang M.S., Yu C.J., Yang P.C., Hsiao M., Hsu T.L., Wong C.H. (2013). Fucosyltransferase 8 as a functional regulator of nonsmall cell lung cancer. Proc. Natl. Acad. Sci. U. S. A..

[15] Tu C.F., Wu M.Y., Lin Y.C., Kannagi R., Yang R.B. (2017). FUT8 promotes breast cancer cell invasiveness by remodeling TGF-beta receptor core fucosylation. Breast Cancer Res..

[16] The human protein Atlas. https://www.proteinatlas.org/ENSG00000033170-FUT8/pathology/endometrial+cancer,2020/01/07.

[17] Wang J.W., Ambros R.A., Weber P.B., Rosano T.G. (1995). Fucosyltransferase and alpha-L-fucosidase activities and fucose levels in normal and malignant endometrial tissue. Cancer Res..

[18] Ma B., Simala-Grant J.L., Taylor D.E. (2006). Fucosylation in prokaryotes and eukaryotes. Glycobiology.

[19] Chachadi V.B., Bhat G., Cheng P.W. (2015). Glycosyltransferases involved in the synthesis of MUC-associated metastasis-promoting selectin ligands. Glycobiology.

[20] Miyoshi E., Noda K., Yamaguchi Y., Inoue S., Ikeda Y., Wang W., Ko J.H., Uozumi N., Li W., Taniguchi N. (1999). The alpha1-6-fucosyltransferase gene and its biological significance. Biochim. Biophys. Acta.

[21] Taniguchi N., Kizuka Y. (2015). Glycans and cancer: role of N-glycans in cancer biomarker, progression and metastasis, and therapeutics. Adv. Canc. Res..

[22] Noda K., Miyoshi E., Uozumi N., Gao C.X., Suzuki K., Hayashi N., Hori M., Taniguchi N. (1998). High expression of alpha-1-6 fucosyltransferase during rat hepatocarcinogenesis. Int. J. Canc..

[23] Taketa K., Endo Y., Sekiya C., Tanikawa K., Koji T., Taga H., Satomura S., Matsuura S., Kawai T., Hirai H. (1993). A collaborative study for the evaluation of lectin-reactive alpha-fetoproteins in early detection of hepatocellular carcinoma. Cancer Res..

[24] Morrow C.P., Bundy B.N., Kurman R.J., Creasman W.T., Heller P., Homesley H.D., Graham J.E. (1991). Relationship between surgical-pathological risk factors and outcome in clinical stage I and II carcinoma of the endometrium: a Gynecologic Oncology Group study. Gynecol. Oncol..

[25] Lanciano R.M., Corn B.W., Schultz D.J., Kramer C.A., Rosenblum N., Hogan W.M. (1993). The justification for a surgical staging system in endometrial carcinoma, Radiotherapy and oncology. J. Eur. Soc. Therapeut. Radiol. Oncol..

[26] Ito Y., Miyauchi A., Yoshida H., Uruno T., Nakano K., Takamura Y., Miya A., Kobayashi K., Yokozawa T., Matsuzuka F., Taniguchi N., Matsuura N., Kuma K., Miyoshi E. (2003). Expression of α1,6-fucosyltransferase (FUT8) in papillary carcinoma of the thyroid: its linkage to biological aggressiveness and anaplastic transformation. Canc. Lett..

[27] Ambros R.A., Kurman R.J. (1993). Association of Ulex europaeus agglutinin I binding with invasion in endometrial carcinoma. Int. J. Gynecol. Pathol..

[28] Aoki D., Nozawa S., Iizuka R., Kawakami H., Hirano H. (1990). Differences in lectin binding patterns of normal endometrium and endometrial adenocarcinoma, with special reference to staining with Ulex europeus agglutinin 1 and peanut agglutinin. Gynecol. Oncol..

[29] Kurimoto A., Kitazume S., Kizuka Y., Nakajima K., Oka R., Fujinawa R., Korekane H., Yamaguchi Y., Wada Y., Taniguchi N. (2014). The absence of core fucose up-regulates GnT-III and Wnt target genes: a possible mechanism for an adaptive response in terms of glycan function. J. Biol. Chem..

[30] Nishikawa A., Ihara Y., Hatakeyama M., Kangawa K., Taniguchi N. (1992). Purification, cDNA cloning, and expression of UDP-N-acetylglucosamine: beta-D-mannoside beta-1,4N-acetylglucosaminyltransferase III from rat kidney. J. Biol. Chem..

[31] Gu J., Nishikawa A., Tsuruoka N., Ohno M., Yamaguchi N., Kangawa K., Taniguchi N. (1993). Purification and characterization of UDP-N-acetylglucosamine: alpha-6-D-mannoside beta 1-6N-acetylglucosaminyltransferase (N-acetylglucosaminyltransferase V) from a human lung cancer cell line. J. Biochem..

[32] Schachter H. (1986). Biosynthetic controls that determine the branching and microheterogeneity of protein-bound oligosaccharides. Biochem. Cell Biol.= Biochimie et biologie cellulaire.

[33] Nishida M., Kasahara K., Kaneko M., Iwasaki H., Hayashi K. (1985). [Establishment of a new human endometrial adenocarcinoma cell line, Ishikawa cells, containing estrogen and progesterone receptors]. Nippon. Sanka Fujinka Gakkai Zasshi.

[34] Liu Y.C., Yen H.Y., Chen C.Y., Chen C.H., Cheng P.F., Juan Y.H., Chen C.H., Khoo K.H., Yu C.J., Yang P.C., Hsu T.L., Wong C.H. (2011). Sialylation and fucosylation of epidermal growth factor receptor suppress its dimerization and activation in lung cancer cells. Proc. Natl. Acad. Sci. U. S. A..

[35] Venkatachalam M.A., Weinberg J.M. (2013). New wrinkles in old receptors: core fucosylation is yet another target to inhibit TGF-beta signaling. Kidney Int..

[36] Wang X., Gu J., Ihara H., Miyoshi E., Honke K., Taniguchi N. (2006). Core fucosylation regulates epidermal growth factor receptor-mediated intracellular signaling. J. Biol. Chem..

[37] Wang Y., Fukuda T., Isaji T., Lu J., Im S., Hang Q., Gu W., Hou S., Ohtsubo K., Gu J. (2015). Loss of alpha1,6-fucosyltransferase inhibits chemical-induced hepatocellular carcinoma and tumorigenesis by down-regulating several cell signaling pathways. Faseb. J..

[38] Shin J.M., Yoo K.J., Kim M.S., Kim D., Baek K.H. (2006). Hyaluronan- and RNA-binding deubiquitinating enzymes of USP17 family members associated with cell viability. BMC Genom..

[39] Ramakrishna S., Suresh B., Bae S.M., Ahn W.S., Lim K.H., Baek K.H. (2012). Hyaluronan binding motifs of USP17 and SDS3 exhibit anti-tumor activity. PloS One.

[40] Matsuo K., Cahoon S.S., Yoshihara K., Shida M., Kakuda M., Adachi S., Moeini A., Machida H., Garcia-Sayre J., Ueda Y., Enomoto T., Mikami M., Roman L.D., Sood A.K. (2016). Association of low-dose aspirin and survival of women with endometrial cancer. Obstet. Gynecol..

[41] Patrignani P., Patrono C. (2018). Aspirin, platelet inhibition and cancer prevention. Platelets.

[42] Xu X.R., Yousef G.M., Ni H. (2018). Cancer and platelet crosstalk: opportunities and challenges for aspirin and other antiplatelet agents. Blood.

[43] Taniguchi H., Yamada T., Takeuchi S., Arai S., Fukuda K., Sakamoto S., Kawada M., Yamaguchi H., Mukae H., Yano S. (2017). Impact of MET inhibition on small-cell lung cancer cells showing aberrant activation of the hepatocyte growth factor/MET pathway. Canc. Sci..

[44] Lee J.J.X., Chan J.J., Choo S.P. (2015). Clinical development of c-MET inhibition in hepatocellular carcinoma. Diseases.

[45] Miwa H.E., Song Y., Alvarez R., Cummings R.D., Stanley P. (2012). The bisecting GlcNAc in cell growth control and tumor progression. Glycoconj. J..

[46] Agrawal P., Fontanals-Cirera B., Sokolova E., Jacob S., Vaiana C.A., Argibay D., Davalos V., McDermott M., Nayak S., Darvishian F., Castillo M., Ueberheide B., Osman I., Fenyo D., Mahal L.K., Hernando E. (2017). A systems biology approach identifies FUT8 as a driver of melanoma metastasis. Canc. Cell.

[47] Yue L., Han C., Li Z., Li X., Liu D., Liu S., Yu H. (2016). Fucosyltransferase 8 expression in breast cancer patients: a high throughput tissue microarray analysis. Histol. Histopathol..

